# Adipose-derived stem cells modulate neuroinflammation and improve functional recovery in chronic constriction injury of the rat sciatic nerve

**DOI:** 10.3389/fnins.2023.1172740

**Published:** 2023-06-29

**Authors:** Szu-Han Chen, Chia-Ching Wu, Wan-Ling Tseng, Fu-I Lu, Ya-Hsin Liu, Shau-Ping Lin, Sheng-Che Lin, Yuan-Yu Hsueh

**Affiliations:** ^1^Division of Plastic and Reconstructive Surgery, Department of Surgery, National Cheng Kung University Hospital, College of Medicine, National Cheng Kung University, Tainan, Taiwan; ^2^Center of Cell Therapy, National Cheng Kung University Hospital, College of Medicine, National Cheng Kung University, Tainan, Taiwan; ^3^International Research Center for Wound Repair and Regeneration, National Cheng Kung University, Tainan, Taiwan; ^4^Institute of Clinical Medicine, College of Medicine, National Cheng Kung University, Tainan, Taiwan; ^5^Department of Cell Biology and Anatomy, College of Medicine, National Cheng Kung University, Tainan, Taiwan; ^6^Department of Biotechnology and Bioindustry Science, College of Bioscience and Biotechnology, National Cheng Kung University, Tainan, Taiwan; ^7^The integrative Evolutionary Galliform Genomics (iEGG) and Animal Biotechnology Center, National Chung Hsing University, Taichung, Taiwan; ^8^Department of Life Sciences, National Cheng Kung University, Tainan, Taiwan; ^9^Institute of Biotechnology, College of Bio-Resources and Agriculture, National Taiwan University, Taipei, Taiwan; ^10^Division of Plastic Surgery, Department of Surgery, An-Nan Hospital, China Medical University, Tainan, Taiwan

**Keywords:** adipose derived stem cells, neuroinflammation, compressive neuropathy, chronic constriction injury, neuropathic pain, stem cell therapy, immunomodulation

## Abstract

**Introduction:**

Compressive neuropathy, a common chronic traumatic injury of peripheral nerves, leads to variable impairment in sensory and motor function. Clinical symptoms persist in a significant portion of patients despite decompression, with muscle atrophy and persistent neuropathic pain affecting 10%–25% of cases. Excessive inflammation and immune cell infiltration in the injured nerve hinder axon regeneration and functional recovery. Although adipose-derived stem cells (ASCs) have demonstrated neural regeneration and immunomodulatory potential, their specific effects on compressive neuropathy are still unclear.

**Methods:**

We conducted modified CCI models on adult male Sprague-Dawley rats to induce irreversible neuropathic pain and muscle atrophy in the sciatic nerve. Intraneural ASC injection and nerve decompression were performed. Behavioral analysis, muscle examination, electrophysiological evaluation, and immunofluorescent examination of the injured nerve and associated DRG were conducted to explore axon regeneration, neuroinflammation, and the modulation of inflammatory gene expression. Transplanted ASCs were tracked to investigate potential beneficial mechanisms on the local nerve and DRG.

**Results:**

Persistent neuropathic pain was induced by chronic constriction of the rat sciatic nerve. Local ASC treatment has demonstrated robust beneficial outcomes, including the alleviation of mechanical allodynia, improvement of gait, regeneration of muscle fibers, and electrophysiological recovery. In addition, locally transplanted ASCs facilitated axon remyelination, alleviated neuroinflammation, and reduced inflammatory cell infiltration of the injured nerve and associated dorsal root ganglion (DRG). Trafficking of the transplanted ASC preserved viability and phenotype less than 7 days but contributed to robust immunomodulatory regulation of inflammatory gene expression in both the injured nerve and DRG.

**Discussion:**

Locally transplanted ASC on compressed nerve improve sensory and motor recoveries from irreversible chronic constriction injury of rat sciatic nerve via alleviation of both local and remote neuroinflammation, suggesting the promising role of adjuvant ASC therapies for clinical compressive neuropathy.

## 1. Introduction

Compressive neuropathy is a common chronic traumatic peripheral nerve injury (Tham et al., 1996; Robertson and Saratsiotis, 2005). Patients experiencing compressive neuropathy often exhibit varying degrees of impairment in sensory and motor function (Assmus et al., 2011). The gold standard treatment is currently complete surgical decompression. Although some patients are initially satisfied with the improvement of clinical symptoms and functional outcomes (Jones et al., 2012), ~10–25% may return with the recurrence of symptoms including paresthesia, neuropathic pain, and weakness (Jones et al., 2012; Tang et al., 2016). There is evidence for the effective alleviation of neuropathic pain for several medications, but they have side effects that may preclude long-term use (Dworkin et al., 2003). Invasive interventional therapy, including neural blockade and electrical stimulation therapy, has shown some benefits, but there is no solid evidence base (Baron et al., 2010). The drawbacks of these interventions for neuropathic pain should be considered, such as wound infection, aseptic meningitis, transient paraparesis, epidural hematoma, epileptic seizures, and skin reactions (Cruccu et al., 2007; Attal, 2012). The etiology of refractory neuropathic pain may include persistent ischemia, prolonged neuroinflammation, and extensive perineural fibrous proliferation (Schmid et al., 2013, 2020; Chen et al., 2021).

Mechanical compression of peripheral nerves breaks down the blood–nerve barrier, resulting in the destruction of homeostasis within the endoneurial environment. Microvessel leakage within the endoneurium increases endoneurial fluid pressure and edema, followed by the infiltration of inflammatory cells such as lymphocytes, fibroblasts, and macrophages. Prolonged neuroinflammation further initiates local and remote neural impairment and subsequent extensive scar formation (Mackinnon, 2002; Richner et al., 2018). Local activated immune cells release inflammatory mediators, including cytokines (e.g., IL-1b and TNF-a), which mediate the recruitment of circulating immune cells, including neutrophils, macrophages, and T cells. Treatments aimed at improving exaggerated segmental demyelination and neuroinflammation will contribute to preventing eventual perineurial fibrosis and persistent neuropathic pain (Ellis and Bennett, 2013).

Stem cell therapy has been extensively applied clinically to various inflammatory and autoimmune diseases (Uccelli et al., 2008). The interactions between stem cells and immune cells contribute to tissue remodeling and healing by regulating inflammatory cytokines (Kim and Hematti, 2009). The immunomodulatory potential of mesenchymal stem cells for preventing excessive tissue damage and promoting tissue repair is well-recognized (Ma et al., 2018; Regmi et al., 2019). Adipose-derived stem cells (ASCs), a type of mesenchymal stem cell, have demonstrated therapeutic benefits for neurological recovery in animals and humans (Rhode et al., 2021; Podsednik et al., 2022). ASCs release a range of neurotrophic factors, such as epidermal growth factor, transforming growth factor-β1, vascular endothelial growth factor, basic fibroblast growth factor, hepatocyte growth factor, insulin-like growth factor, and brain-derived neurotrophic factor, at different stages of tissue regeneration. They are also believed to promote the healing of damaged peripheral nerves (Zack-Williams et al., 2015; Wang et al., 2019). Despite therapeutic evidence of the neurotrophic and immunomodulatory effects of ASC cell therapy for peripheral nerve regeneration, there is currently no reported evidence for the effects of such therapy on compressive neuropathy. In this study, we aimed to investigate the therapeutic effects of locally delivered ASCs on the modulation of neuroinflammation and the functional recovery of compressive neuropathy.

## 2. Materials and methods

### 2.1. Animals and surgical procedures

Animal protocols and surgical procedures, such as animal housing and care, were approved by the Laboratory Animal Center and Institutional Animal Care and Use Committee (IACUC, No. 109250) of the National Cheng Kung University. In this study, 28 Sprague–Dawley female rats weighing 125–150 g were used. The average operation time for each animal was ~15 min under general anesthesia induced by the inhalation of 3% isoflurane (USP, Sigma-Aldrich, St. Louis, MO) followed by 1.5–2% maintenance dosage.

All rats underwent two procedures. First, a modified chronic constriction injury (CCI) was induced in the left sciatic nerve according to the instructions described in our previous studies (Chen et al., 2020, 2021). In brief, the left sciatic nerve was exposed at the mid-thigh level. The area proximal to the trifurcation of the sciatic nerve was freed from the adhering tissue. Four ligatures (5-0 Nylon, Ethicon US, Bridgewater, NJ) were tied around the sciatic nerve at ~1 mm intervals. Controllable constriction forces on the rat ipsilateral sciatic nerve were judiciously applied with computer monitoring (6 g string tension). After 7 days of constriction, reproducible peripheral neuropathy is induced, causing significant mechanical allodynia, moderate muscle atrophy, and increased neuroinflammatory signals, as described in our previous study (Chen et al., 2020). The CCI rats underwent secondary operation 7 days after the first surgical procedure. The injured left sciatic nerve was exposed, and the adherent connective tissue and nylon ligatures were carefully removed. After functional assessment for 1 month, all rats were euthanized according to standard IACUC-approved procedures. The bilateral hindlimb gastrocnemius muscles, sciatic nerves, and DRGs were harvested for further analysis.

After the nylon ligatures were removed, for the ASC group, the 1 × 10^6^ ASCs suspended in 50 μl of phosphate-buffered saline (PBS) were injected smoothly through a 24 G needle into the epineurium of the injured sciatic nerve under a microscope according to the previously described methods (Rodriguez Sanchez et al., 2019; Jiang et al., 2022). In the control group, 50 μl of PBS buffer solution was injected. After injection, the surrounding connective tissue and skin were repaired using 3-0 Nylon (Ethicon US, Bridgewater, NJ). Postoperatively, all animals were checked daily for normal behavior and signs of infection, distress, pain (e.g., autotomy), or weight loss.

### 2.2. Isolation, cell culture, and flow cytometry analysis of ASCs

ASCs from healthy donors were obtained with informed consent and approved according to the procedures of the institutional review board of National Cheng Kung University Hospital (NCKUH IRB No.A-ER-109-127). The ASC isolation protocols were established by Dr. Zuk and Dr. Hedrick at UCLA (Zuk et al., 2001). The multipotency of isolated ASCs was assessed by performing osteogenic, adipogenic, and chondrogenic induction, as described (Hsueh et al., 2012). Briefly, the raw lipoaspirates were washed with sterile PBS to remove contaminating debris and red blood cells (RBCs) and treated with 0.075% collagenase (type I; Sigma-Aldrich, St. Louis, MO) in PBS for 30 min at 37°C with gentle agitation. The collagenase was inactivated with an equal volume of Dulbecco's modified Eagle's medium (Invitrogen Inc., Carlsbad, CA) with 10% fetal bovine serum (HyClone, Logan, UT). The infranatant was centrifuged for 10 min at low speed, separating the pelleted stromal vascular fraction (SVF) from the floating mature adipocytes. The SVF consisted of a heterogeneous cell population and was enriched for the preadipocyte population by using plastic adherence. All SVF cells were cultured and passaged in DMEM containing 10% FBS at least three times before use. In passage 3, isolated single ASCs were harvested, and the surface markers were stained with the monoclonal antibodies as described in the following list. Surface markers of ASCs (CD34, CD90, and CD105) were detected for the fourth passage of ASCs by flow cytometry (Table 1). The ASCs were incubated for half an hour with monoclonal antibodies at 1:200 in PBS and acquired on a CytoFLEX Flow Cytometer (Beckman Coulter, USA). Detailed information on ASC identification is shown in Supplementary Table 1.

**Table 1:** Representative surface markers of ASCs.

**Protein**	**Catalog number**	**Brand**	**Dilution**
Mouse anti-human CD34	Ab54208	Abcam	1:100
Mouse anti-human CD90	14-0909-82	Invitrogen	1:100
Rabbit anti-human CD105	Ab231774	Abcam	1:100

### 2.3. The trilineage differentiation of cultured ASCs *in vitro*

The ASCs were taken from the incubator and carefully aspirated into the medium and washed 2–3 times with Dulbecco PBS, and the plate was transferred to a fume hood. The cellular monolayer was covered with a fixative solution. After at least 10 min, the fixative solution was aspirated and the ASCs were washed with distilled water and then 50 μl of Oil Red O/Alizarin Red S/Alcian blue staining solution was added to cover the cellular monolayer. This cellular monolayer was incubated at room temperature in the dark for 45 min. The staining solution was aspirated, and the cellular monolayer was washed two times with PBS. Another 100 μl of PBS was added, and then, the cells were inspected under a light microscope and their images were captured. Undifferentiated ASCs (without extracellular lipid droplets) are white, whereas ASC-derived lipocytes (with extracellular lipid droplets) are bright orange-red.

### 2.4. ASC labeling and tracking

The indocyanine green (ICG) solution used in this study was prepared by dissolving ICG powder (Carbosynth Limited, UK) in alpha minimum essential medium (αMEM, Gibco) to give a stock solution of 2.5 mg/mL. For ICG labeling, 1 × 10^6^ ASCs were incubated at 0.5 mg/ml of ICG solution for 30 min at 37°C. After labeling, the cells were washed three times with DPBS and further cultured in complete αMEM. ASCs were injected directly into the compressed sciatic nerve, and each rat was imaged every 2 days for 9 days until the fluorescence signal matched that of the PBS control. *In vivo* fluorescence imaging was performed with a Xenogen IVIS^®^ Spectrum Noninvasive Quantitative Molecular Imaging System (PerkinElmer Inc. Waltham, MA). All images were acquired using an excitation wavelength of 745 nm and an emission wavelength of 820 nm. Following the acquisition, all images were normalized to the units of the average efficiency and displayed on the same scale of fluorescence intensity. Bioluminescence from the region of interest was manually delineated, and the data were expressed as photon fluxes (photons/s/cm^2^/steradian). The bioluminescence data were collected and analyzed using the IVIS^®^ Living Image System.

### 2.5. Sensory assessment

Mechanical allodynia was measured by the von Frey test for the direct measurement of the threshold force for paw withdrawal forces. Spontaneous foot lifting may provide a behavioral measure of spontaneous pain. The rats stood on an elevated wide-gauge wire mesh platform. From below, a von Frey hair was poked through the mesh to the undersurface of a hind paw. At the threshold, the animal would respond by flicking its paw away from the hair. The withdrawal forces of the rat hindlimb were measured by the von Frey test on days −7, 0, 7, 14, and 28 and analyzed.

### 2.6. Gait analysis for functional outcome evaluation

For assessing motor nerve recovery, motion gait analysis was performed as described in previous reports with minor modifications (Hsueh et al., 2014). All animals were gently handled and tested in a quiet environment to minimize stress levels. Before the test, the rats were allowed conditioning trials on a 6 × 80 cm walking track, and the image processing was performed using MATLAB software. The sciatic function index (SFI) was obtained according to the following formula using print length, toe spread, and intermediary toe spread.


$$\begin{array}{l} \text{SFI}=38.3(\text{EPL}-\text{NPL})/\text{NPL}+109.5(\text{ETS}-\text{NTS})/\text{NTS}\\ \;+13.3(\text{EIT}-\text{NIT})/\text{NIT}-8.8 \end{array}$$

The maximum value for each measurement was used, and the data were analyzed with RatMotion_Gait_Software. The SFI, an indicator of the degree of nerve dysfunction, varied from 0 (normal) to −100 (complete dysfunction). Three footprints of each rat were analyzed by a single observer, and the average of the measurements was used in the SFI calculations. All rats were tested on days 0, 7, 14, and 28 after ASC therapy.

### 2.7. Electrophysiological assessment

The rats were tested at week 4 after ASC therapy. The rats were anesthetized with 3% isoflurane by inhalation and laid on their right side on an electrically heated pad in a warm room. The exposed hindquarters and the left leg were covered with a jacket made from a parallel array of silicone rubber tubes through which circulated water was maintained at 37°C. Recordings were made only when rectal and skin temperatures over the thigh and ankle were all at 37 ± 0.5°C. The animals were warmed if they were below this temperature at the start of the experiment. Before harvesting muscle samples, the compound muscle action potentials (CMAPs) of each muscle were measured after stimulating the sciatic nerve in the hindlimbs using needle electrodes as previously described. The rat sciatic nerve was exposed, and a single-pulse shock (0.2 mA, 0.2 ms) was applied to the sciatic nerve trunk. A concentric needle electrode was inserted into the foot muscles. The sciatic nerve was stimulated through a needle electrode at the sciatic notch. The evoked muscle action potentials were amplified and displayed on the nerve conduction velocity (NCV) device monitor. The evoked action potential of individual axons was measured using a characteristic spike waveform and conduction velocity, which was dependent on the diameter of the nerve fiber or the condition of nerve myelination (Navarro and Udina, 2009; Werdin et al., 2009). The latency of the compound muscle action potential (CMAP) determined the conduction time of the impulse along the nerve and the transmission time across the neuromuscular junction. CMAPs were recorded on the gastrocnemius muscle belly at 10 mV/5 ms. Normal CMAPs from the contralateral side of the sciatic nerve were recorded for comparison. The Nihon Kohden Neuropack^®^ X1 MEB-2300 EMG/NCV/EP Measuring Desktop System (Japan Inc.) was used for the test and data acquisition.

### 2.8. Wet muscle ratio evaluation and histological analysis

The gastrocnemius muscle, the largest muscle innervated by the sciatic nerve in rats, starts to atrophy after nerve injury. To assess nerve re-innervation, the gastrocnemius muscle weight and fiber diameters were measured immediately after the rats were euthanized. The gastrocnemius muscles (excluding the soleus muscles) of both limbs were separated from their bony attachments. Immediately after measuring the muscle weight, the muscle tissues were fixed and the nerve tissue histological assessment was performed. H&E staining was used to examine the muscle fiber morphology.

### 2.9. Immunofluorescent staining

Several histological stains were performed to observe the tissue morphology and protein expression. Immunofluorescent (IF) staining was performed to detect specific expression patterns exhibited by proteins. The primary antibodies used were NF200 (N0142, 1:200; Sigma-Aldrich, St. Louis, MO, USA); s100 beta (ab52642, 1:200), CD68 (ab125212, 1:200), and TNF-α (ab205587, 1:200) from Abcam, Cambridge, MA, USA; and IL-1β (GTX74034, 1:200) and IB4 (GTX54372) from GeneTex, Irvine, CA, USA. The tissue was embedded in OCT (Tissue-Tek^®^, Sakura Finetek Inc, Torrance, CA, USA) and deep-frozen until use. The nerves were cryo-sectioned at 10 μm and mounted in Mowiol^®^ (Sigma-Aldrich, St. Louis, MO, USA). The slices were visualized and photographed with fluorescence microscopy (BX61, Olympus, Tokyo, Japan).

### 2.10. Quantitative PCR of nerve and dorsal root ganglion

The total RNA was extracted from the nerves and DRG tissues of rats using TRIzol reagent (Sigma-Aldrich, St. Louis, MO, USA) according to the manufacturer's instructions. The total RNA was precipitated with 2-propanol, washed twice with 75% ethanol, and resuspended in diethylpyrocarbonate (DEPC)-treated water. The total RNA concentration was determined by measuring the optical density values of the samples at 260 nm. To prepare first-strand cDNA, mRNA was reverse-transcribed with the reverse transcriptase enzyme (ImProm-II™ Reverse Transcriptase, Promega, Madison, WI, USA) in a reaction mixture (20 mL in total). Primers were designed for CD11b, CD68, TNF-α, IL-1β, CD86, CD80, vasoactive intestinal polypeptide (VIP), Substance P, and glyceraldehyde 3-phosphate dehydrogenase (GAPDH) (Table 2). GAPDH was used as a reference gene. Quantitative PCR was performed to analyze the mRNA levels, using the SYBR PCR Master Mix (GoTaq^®^ Green Master Mix, ProMega, Madison, WI, USA) and StepOnePlus™ System (Thermo Fisher Scientific, Waltham, MA, USA). The quantitative PCR conditions were as follows: 10 min at 95°C, followed by 40 cycles of 15 s at 95°C and 30 s at 60°C. The 2–ΔΔCT method was used to analyze the quantitative PCR data.

**Table 2:** Primer sequences and information in this study.

**Gene**	**Sequence (5^′^ → 3^′^)**	**Amplicon (bp)**	**Accession number**
iNOS	F:AATCTTGGAGCGAGTTGTGG	139	XM_039085203.1
	R:CAGGAAGTAGGTGAGGGCTTG		U26686.1
GAPDH	F:TGGCCTCCAAGGAGTAAGAA	129	XM_039107008.1
	R:TGTGAGGGAGATGCTCAGTG		
CD68	F:CTGTTGCGGAAATACAAGCA	144	XM_032914349.1
	R:GGCAGCAAGAGAGATTGGTC		NM_001031638.1
CD80	F:TTCAGACAGGGGCACATACA	173	U88622.1
	R:CGGAAGCAAAGCAGGTAATC		XM_039088035.1
CD86	F:CCTCCAGCAGTGGGAAAC	135	XM_006248399.4
	R:GTAGGTTTCGGGTATCCTTGC		XM_032900176.1
CD163	F:GGAGCAGATCTGGAACTTCG	164	XM_006237352.4
	R:GTTTGAAGTGCAGAGCCACA		NM_001107887.1
CD206	F:GTTCCGGTTTGTGGAGCAG	99	NM_001106123.2
	R:CGTTTGCATTGCCCAGTA		XM_032885181.1
Cox-2	F:GATTGACAGCCCACCAACTT	199	NM_017232.4
	R:CGGGATGAACTCTCTCCTCA		AF233596.1
TNF-a	F:TACTCCTCAGAGCCCCCAAT	112	NM_012675.3
	R:TCGTGTGTTTCTGAGCATCG		AY427673.1
IL-1-b	F:CTGTGACTCGTGGGATGATG	139	M98820.1
	R:TCCATTGAGGTGGAGAGCTT		NM_031512.2
Caspase 3	F:CCGACTTCCTGTATGCTTACTC	184	NM_012922.2
	R:CAGGGAGAAGGACTCAAATTC		
Bcl-2	F:ACTCTTCAGGGATGGGGTGA	93	NM_016993.1
	R:TGACATCTCCCTGTTGACG		
CD11b	F:TCAGTTGTCCGAGCCTTCTT	117	NM_012711.1
	R:TGTCCACACAGTCCGGTAAA		
substance P	F:TCCGACAGTGACCAAATCAA	141	AH002233.2
	R:CTTGTGCTTTGTCCGGGTAT		
VIP	F:CAGAAGCAAGCCTCAGTTCC	113	BC158798.1
	R:GCCTGTCATCCAACCTCACT		

### 2.11. Statistical analysis

General statistical analysis was used for experiment design and data analysis. The sample size necessary to detect a significant effect was estimated using Power and Precision statistical software (Englewood, NJ) with the following information: minimum significant effect to be detected, data variation, power (0.8), and Type I error rate (0.05). The Student's *t*-test was used for two-sample comparisons. For multiple-sample comparison, analysis of variance (GraphPad Prism, ver. 9.4.0) was performed to detect whether a significant difference existed between groups with different treatments, and a multiple comparison procedure Holm's *t*-test was used for post-analysis to find where the differences existed. A *p*-value of 0.05 or less indicated a significant difference between samples.

## 3. Results

### 3.1. ASCs improved sensory and motor recovery of peripheral compressive neuropathy

**Figure 1:**
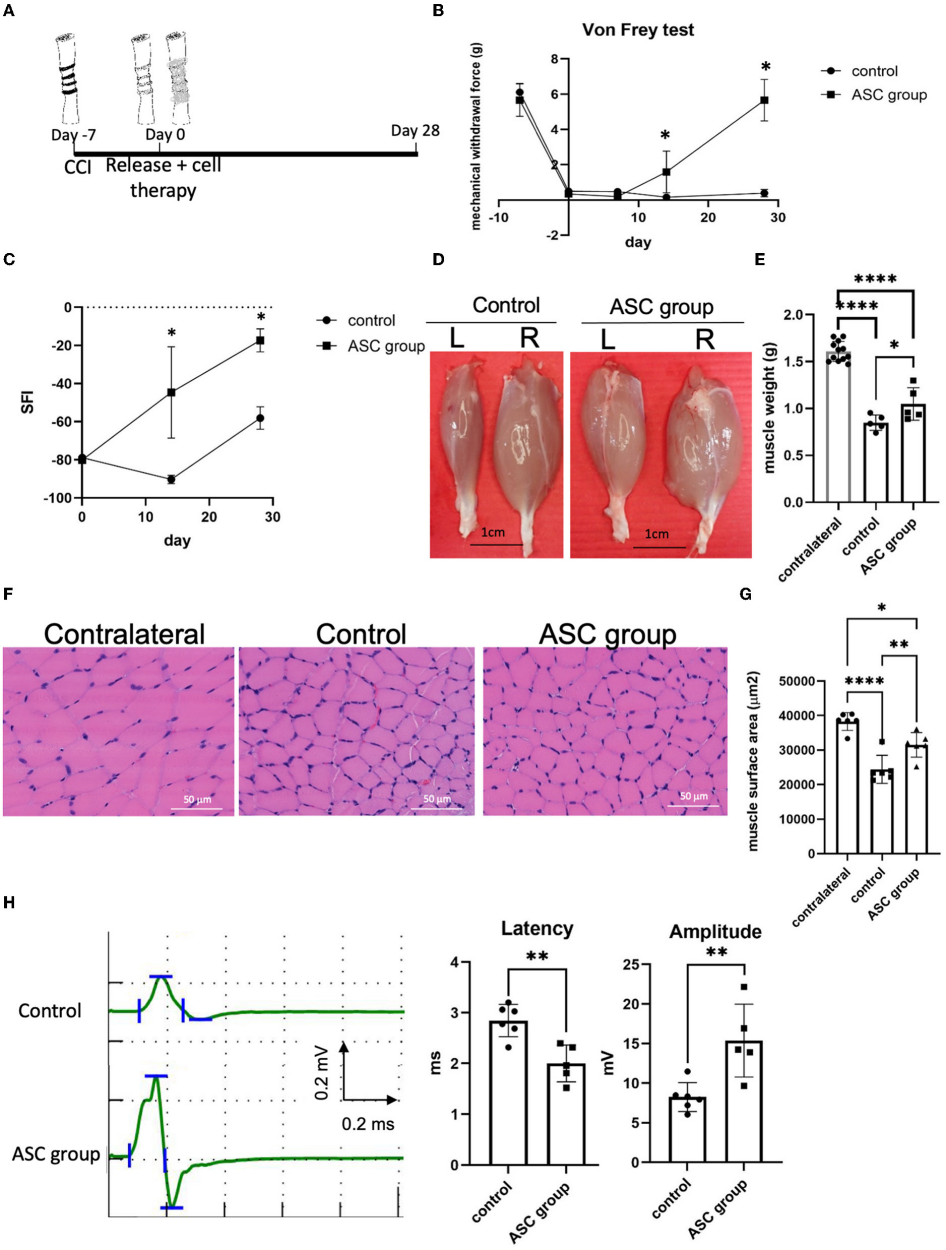
Stem cell therapy promoted sensory and motor functional recoveries from chronic constriction injury of the rodent model. **(A)** Treatment algorism of chronic constriction injury over the ipsilateral sciatic nerve. After 1 week of nerve constriction, one group received nerve release alone plus intraneural PBS injection (as the control group) and the other group received nerve release combined with intraneural stem cells injection (as ASC group). **(B)** Mechanical allodynia was evaluated by von Frey test over the hind paw of rats. The mechanical withdrawal forces (g) indicate the forces that induced paw withdrawal by the von Frey test. After treatment, the ASC group showed increased mechanical withdrawal forces compared with the control group. **(C)** Dynamic gait analysis in the different groups. The ASC group revealed a significantly increased sciatic functional index (SFI)compared with the control group. **(D, E)** Gastrocnemius muscle recovery in the different treatment groups on post-treatment day 28 (L, left injured side; R, right uninjured side). Left gastrocnemius muscle weight demonstrated a significant increase in the ASC group compared with the control group (scale bar: 1 cm) **(F, G)** Histological analysis of left gastrocnemius muscle fiber demonstrated a decrease of muscle fiber surface area in the control group, with an increase in the ASC group on day 28 (Scale bar: 50 mm). **(H)** The electrophysical evaluation of gastrocnemius muscle re-innervation on day 28. The amplitude compound muscle action potential significantly increased in the ASC group compared with the control group, while the latency of nerve conduction was shorter in the ASC group than in the control group (data were presented with mean ± standard deviation, *indicated *p* < 0.05, **indicated *p* < 0.01, ****indicated *p* < 0.0001; *n* = 5 for each group).

Persistent mechanical allodynia with moderate muscle atrophy was reproducibly induced 1 week after the constriction injury, followed by nerve decompression and immediate ASC cell injection into the compressed nerve (Figure 1A). Before intraneural injection, the ASC was verified by cell surface markers of CD90+, CD105+, and CD34– by flow cytometry (Supplementary Figure 1). In addition, multipotent potentials of ASCs were confirmed by trilineage differentiation to fat, cartilage, and bone by oil red, Alcian blue, and alizarin red staining, respectively (Supplementary Figure 1). Subsequent von Frey tests over the left affected hind limb revealed a significant decrease in withdrawal forces on both the control and ASC groups at day 0, compared to day −7. After nerve decompression on day 0, the withdrawal forces of the ASC group had significantly increased by day 14 and returned to baseline at day 28 (Figure 1B). By contrast, the withdrawal forces remained unchanged after nerve decompression from days 0 to 28. Regarding functional sensory-motor coordination, from day 14, the SFI of the ASC group was significantly increased compared to the control group (Figure 1C).

### 3.2. ASCs preserved innervated muscle mass and enhanced neuromuscular re-innervation

After nerve compression, the innervated gastrocnemius muscle of both groups showed a significant decrease in muscle weight on day 28 after nerve decompression (Figures 1D, E). However, the wet weight of the injured gastrocnemius muscle was significantly greater in the ASC group than in the control group on day 28. In addition, the histological examination of the gastrocnemius muscle revealed that the surface area of individual muscle fibers was significantly greater in the ASC group than in the control group (Figures 1F, G). To further investigate the functional re-innervation of the target muscle after CCI, electromyography was performed on day 28 after nerve decompression (Figure 1H). The latency of nerve conduction velocity in the ASC group was significantly shorter than that in the control group. Moreover, the amplitude of CMAP was significantly higher in the ASC group than in the control group.

### 3.3. ASCs facilitate functional axon regeneration and reduce neuroinflammation in compressive neuropathy

**Figure 2:**
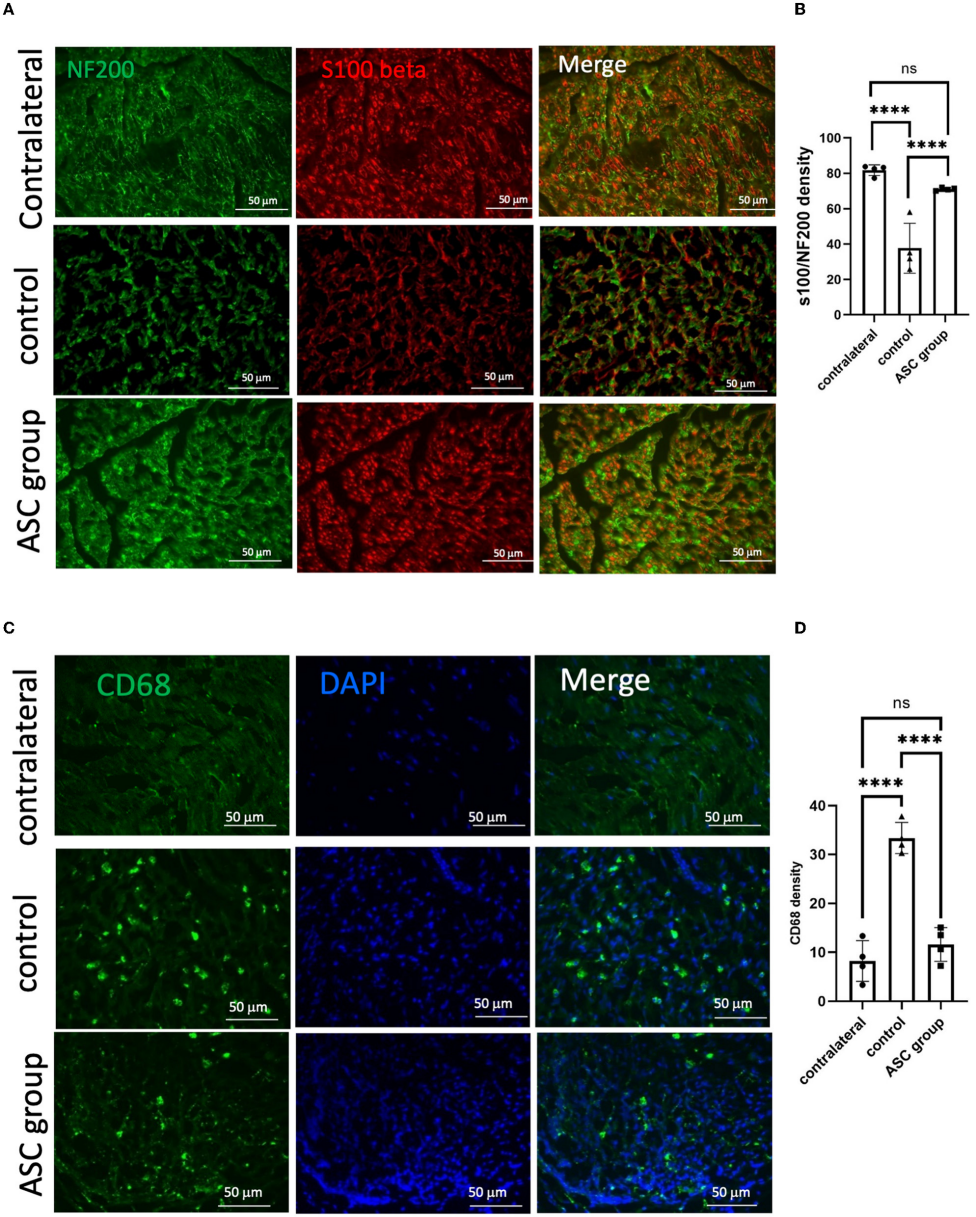
Immunofluorescent staining of functional axon regeneration and inflammatory cell infiltration in the injured nerve at day 28. **(A, B)** Double staining of NF200/S100β revealed decreased regenerated axon density in the control group but increased density in the ASC group. **(C, D)** Inflammatory cell staining with CD68 revealed a significant increase in the control group but a decrease in the ASC group (scale bar: 50 μm; ****indicated *p* < 0.0001, ns indicated no significant difference; *n* = 4 for each group).

**Figure 3:**
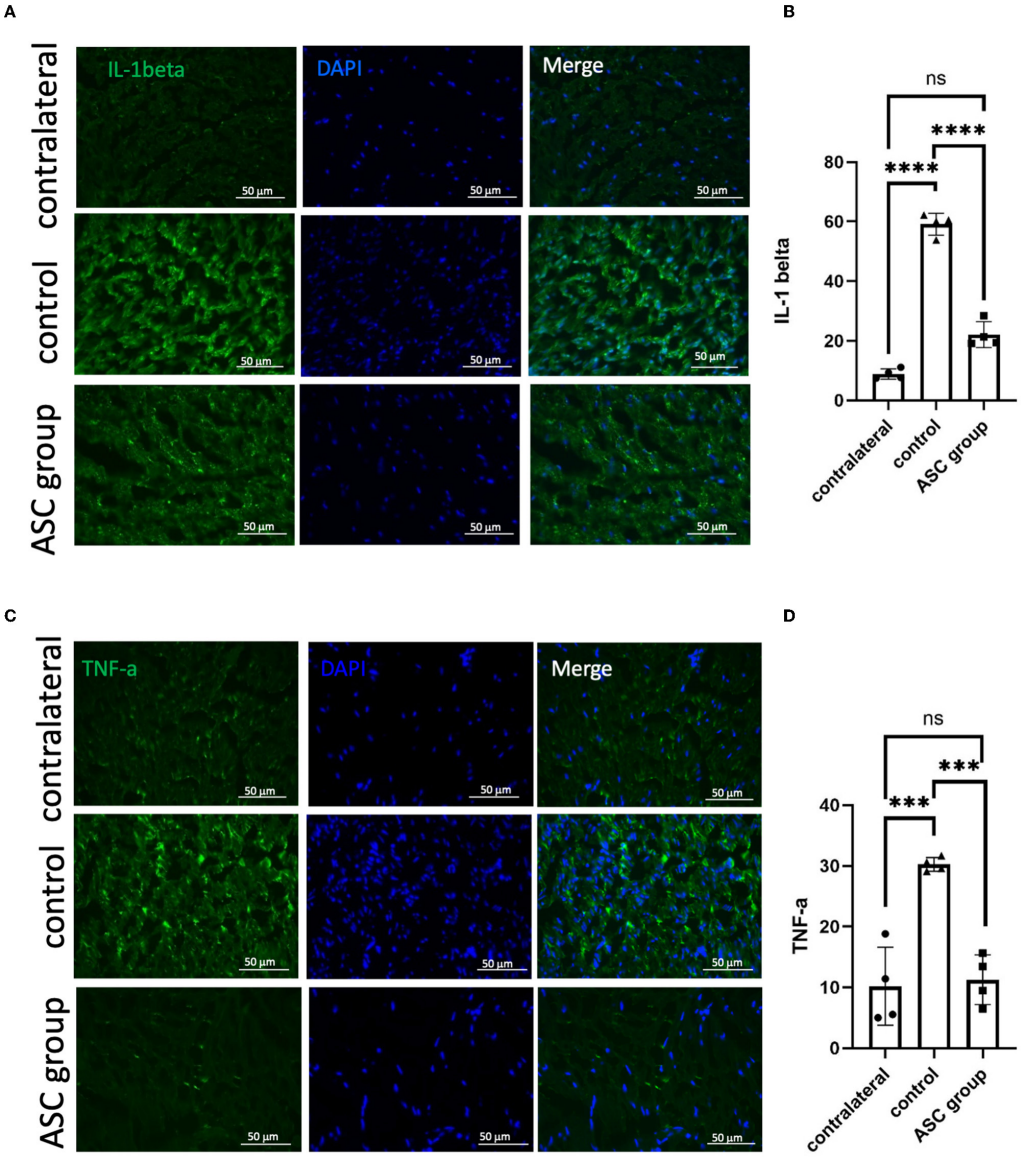
Immunofluorescent staining of neuroinflammatory signals in the injured sciatic nerve at day 28. A significant increase in the signal intensity of TNF-α **(A, B)** and IL-1β **(C, D)** was observed in the control group, as compared to the contralateral nerve as the baseline on day 28. In the ASC group, the signal intensity significantly decreased as compared to the control group (scale bar: 50 μm; ***indicated *p* < 0.001, ****indicated *p* < 0.0001, ns indicated no significant difference; *n* = 4 for each group).

To further explore the beneficial effect of ASC treatment for CCI, histological examination was used to evaluate functional axon regeneration and inflammatory cell infiltration in the compressed nerve on day 28. Immunofluorescent signals of NF200 and S100 at the nerve injury site revealed a significant increase in injured nerves in the ASC group compared with the control group (Figures 2A, B). The neuroinflammatory profile was investigated by the immunofluorescent staining of the compressed sciatic nerve, which revealed a significant increase in CD68+ inflammatory cells in the control group (Figures 2C, D). The number of CD68+-infiltrated cells was significantly decreased in the ASC group. Moreover, the immunofluorescent signals of TNF-α and IL-1β were significantly greater in the control group than in the ASC group (Figure 3). The signal intensity of TNF-α and IL-1β in the ASC group showed no difference compared to the contralateral sciatic nerve.

### 3.4. ASCs reduce pain neuropeptides and neuroinflammatory signals in the ipsilateral DRG

**Figure 4:**
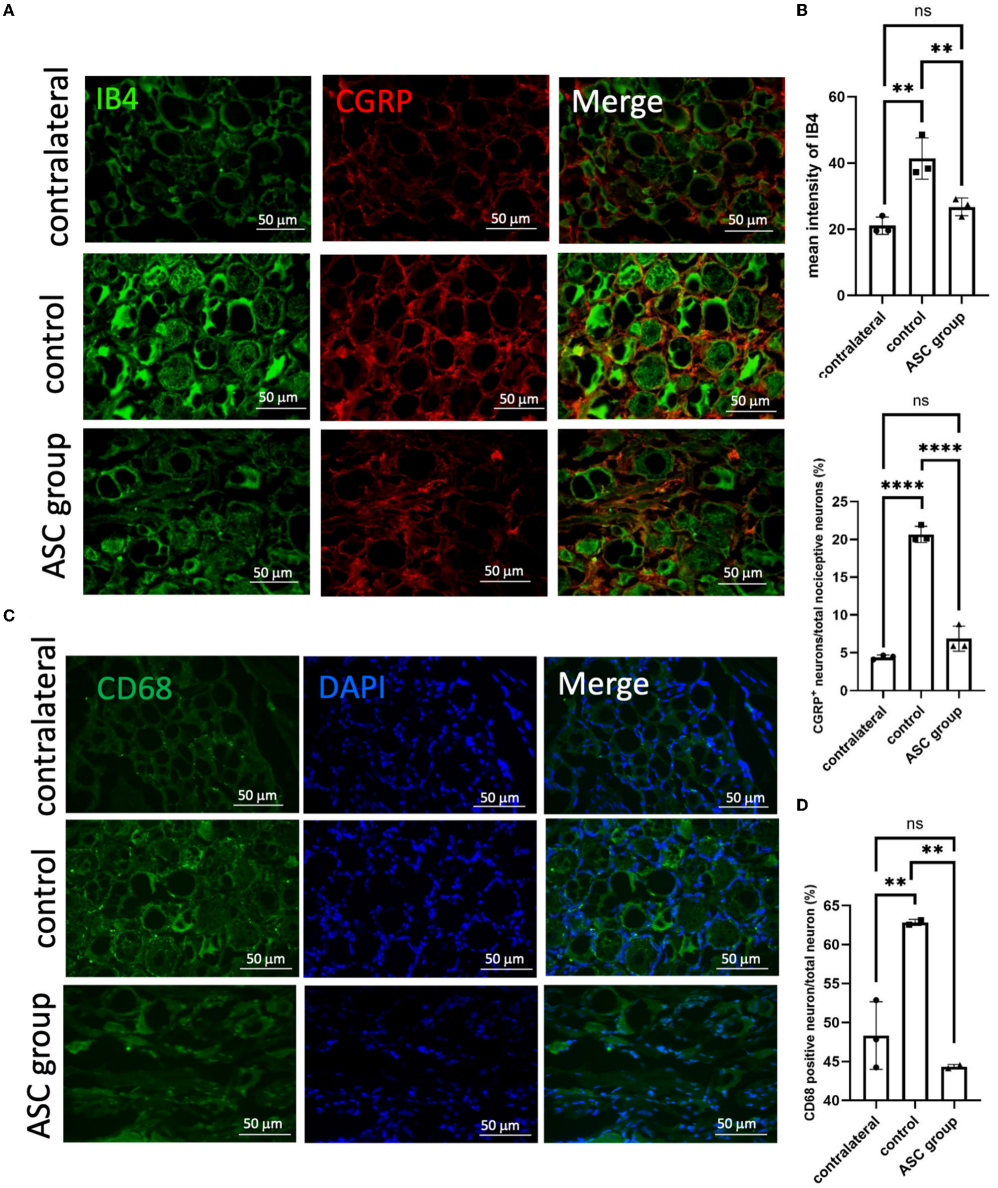
Immunofluorescent staining of pain signals and inflammatory cell infiltration in the injured dorsal root ganglion (DRG) at day 28. A significant increase of signal intensity in IB4 and CGRP **(A, B)** was observed in the control group, as compared to the contralateral DRG as the baseline. In the ASC group, the signal intensity of both IB4 and CGRP significantly decreased as compared to the control group. **(C, D)** Inflammatory cell staining with CD68 revealed a significant increase in the control group but a decrease in the ASC group of injured DRG at day 28 (scale bar: 50 μm; **indicated *p* < 0.01, ****indicated *p* < 0.0001, ns indicated no significant difference; *n* = 3 for each group).

**Figure 5:**
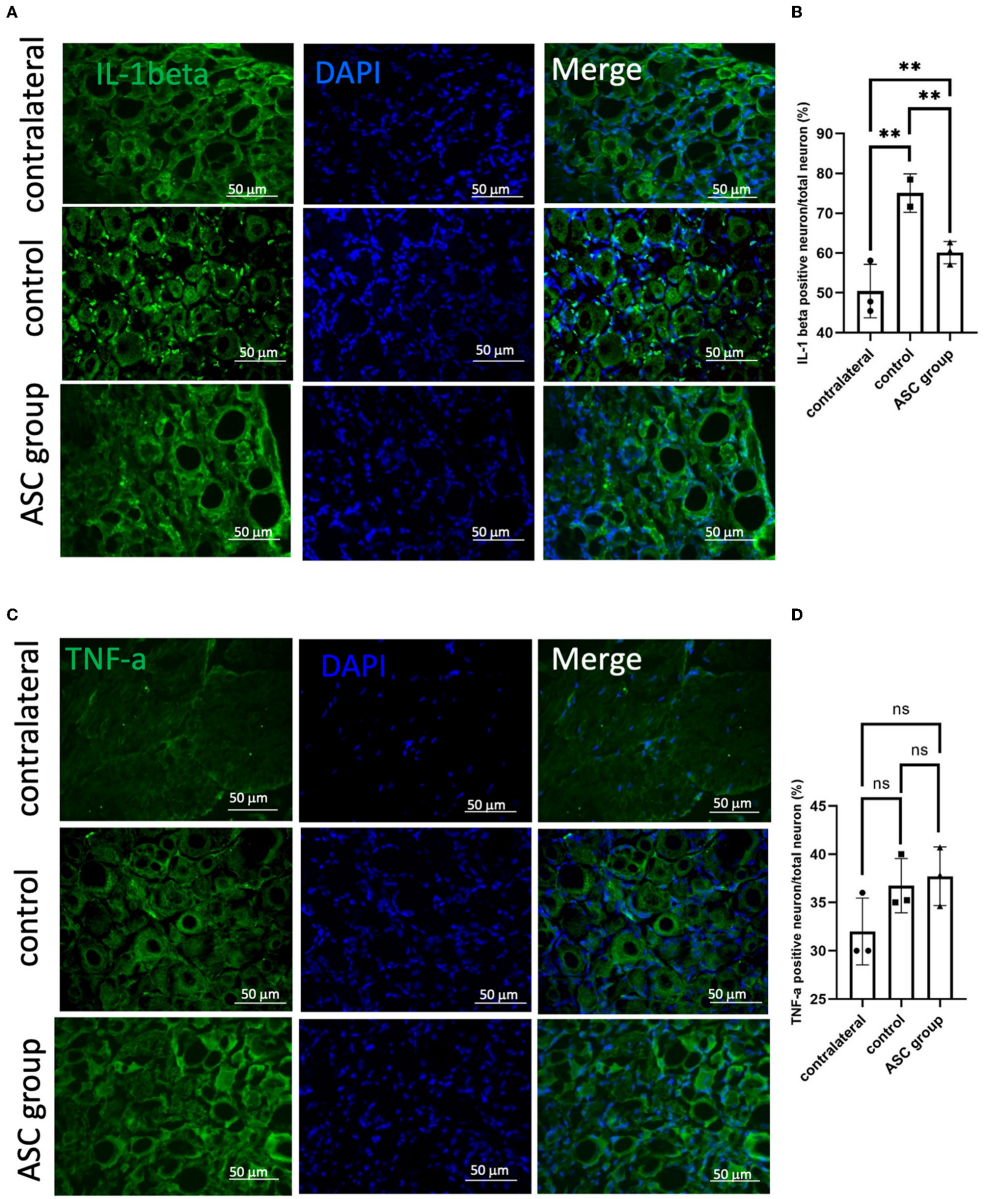
Immunofluorescent staining of neuroinflammatory signals in the injured dorsal root ganglion at day 28. **(A, B)** A significant increase of IL-1β signal was observed in the control group, as compared to contralateral uninjured DRG as a baseline. In the ASC group, the signal intensity significantly decreased as compared to the control group on day 28. **(C, D)** The signal intensity of TNF-α, the other kind of neuroinflammation marker, showed no significant difference between groups (scale bar: 50 μm; **indicated *p* < 0.01, ns indicated no significant difference; *n* = 3 for each group).

To further explore the modulation of neuropathic pain by ASC local treatment, pain-related neuropeptides and neuroinflammatory signals were analyzed at the L3–5 DRG on day 28 after cell therapy. In the injured side of the DRG, pain-related molecules including isolectin B4 (IB4) and calcitonin gene-related peptide (CGRP) were highly expressed in the control group, while they were significantly lower in the ASC group (Figures 4A, B). In addition, the immunofluorescent staining revealed increased CD68+ cells in the control group, but the number of these cells was significantly less in the ASC group (Figures 4C, D). The inflammatory cytokine, IL-1β in the injured side of the DRG, also revealed high expression in the control group, but it was lower in the ASC group (Figures 5A, B). By contrast, the signal intensity of another inflammatory marker, TNF-α, remained unchanged in the control and ASC groups and in the naïve uninjured nerve (the contralateral group) (Figures 5C, D).

### 3.5. Terminal fate of transplanted ASCs

**Figure 6:**
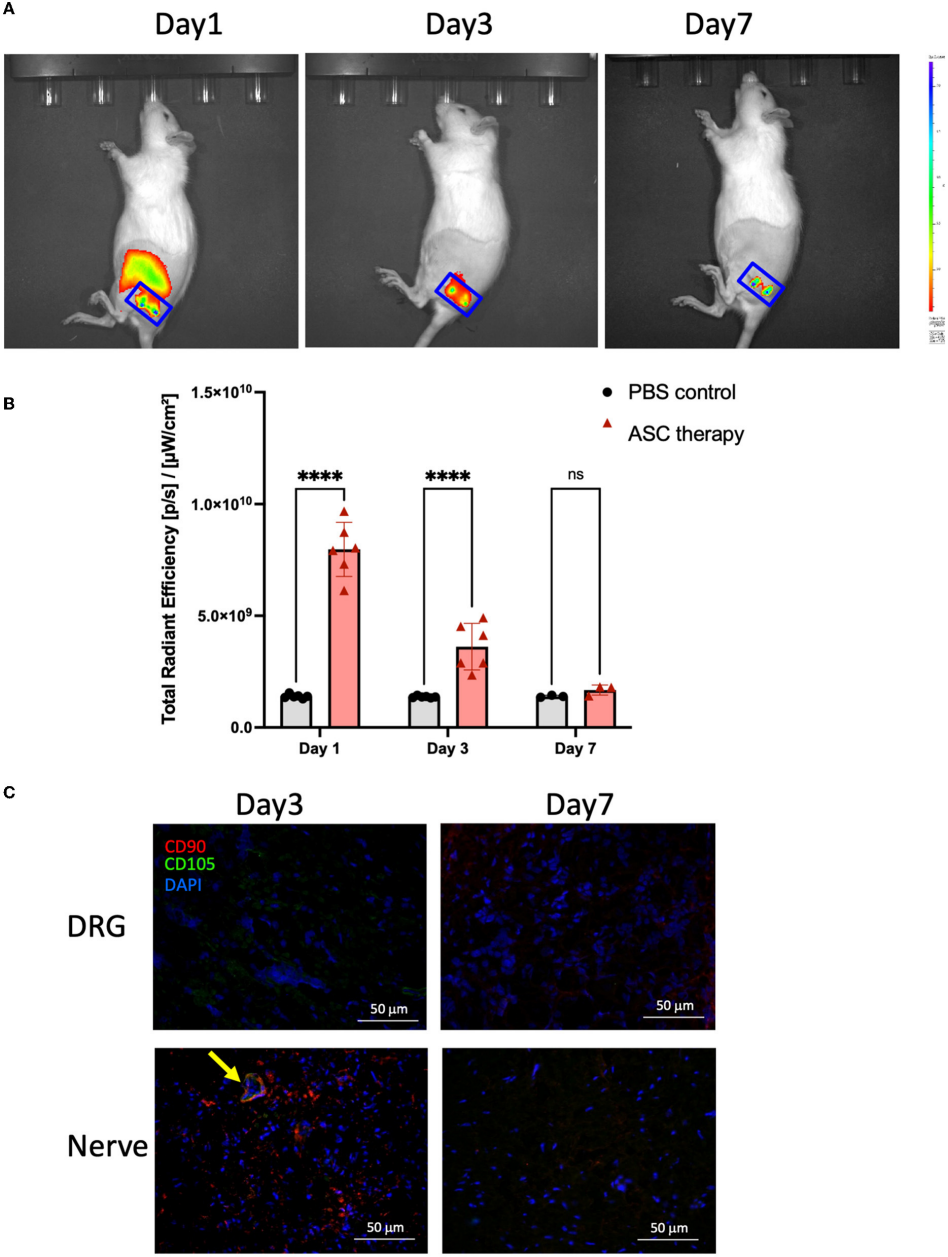
Trafficking for the terminal fate of transplanted ASC after local intraneural injection. **(A, B)** The signal intensity of ICG-bioluminescent labeling ASC was trackable and revealed a significant increase as compared to baseline (PBS injection) on day 3 but returned to baseline signal on day 7. **(C)** Immunofluorescent staining of the injured nerve demonstrated the colocalization of CD90^+^/CD105^+^ cells (arrow), indicating the maintenance of ASC phenotype at least on day 3 (scale bar: 50 μm; ****indicated *p* < 0.0001, ns indicated no significant difference; *n* = 3–5 for each group).

To investigate the potential beneficial mechanism of transplanted ASCs into compressed nerves, cell tracking of transplanted ASCs was performed by *in vitro* cell labeling with ICG. The non-invasive IVIS Spectrum System could track labeled bioluminescent ASCs *in vitro* for more than 7 days (Supplementary Figure 2). After transplantation (day 0), the ASC cell viability was continuously tracked on top of the nerve injection site (Figure 6A). The bioluminescent image of the compressed sciatic nerve demonstrated a significant signal increase in the ASC group on days 1 and 3 compared with the control group (Figure 6B). The signal had declined to baseline intensity with no significant difference between the ASC and control groups by day 7. To confirm ASC cell survival after transplantation, immunofluorescent staining of the transplanted nerve showed the expression of human-specific mesenchymal cell surface antigens CD90+ and CD105+ co-localized in the transplanted nerve on day 3 (Figure 6C). For the ipsilateral DRG, there was no CD90+ or CD105+ cell staining on either days 3 or 7.

### 3.6. Early immunomodulatory effects of transplanted ASCs on injured nerves and DRG

**Figure 7:**
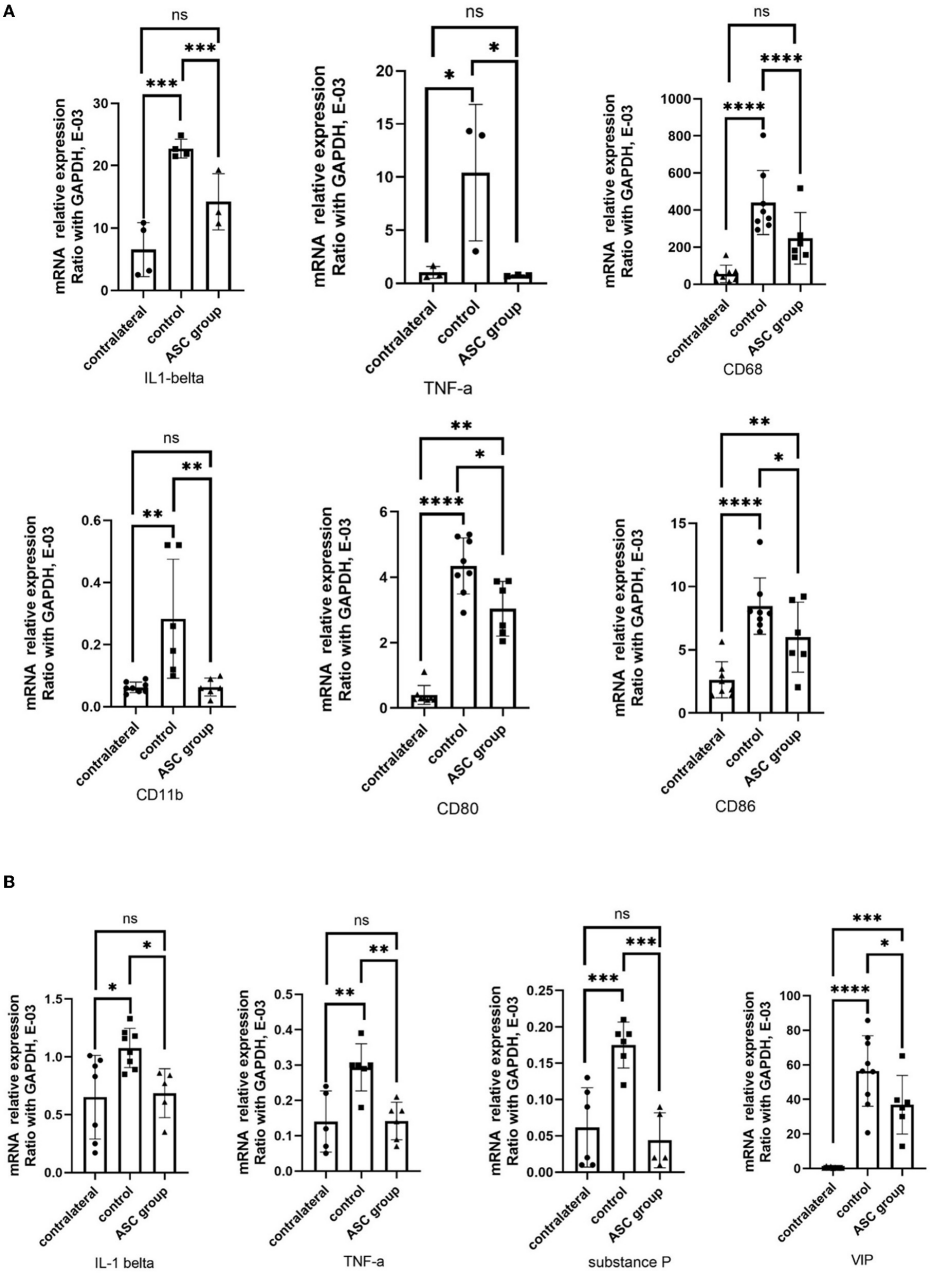
Quantitative PCR analysis of inflammatory gene in the injured nerve dorsal root ganglion at day 3. **(A)** Upregulation of IL-1β, TNF-α, CD68, CD11b, CD80, and CD86 expression in the injured control nerve, as compared to the contralateral uninjured nerve. In the ASC group, significant downregulation of the inflammatory gene expression on the injured nerve as compared to the control group. **(B)** Upregulation of TNF-α, IL-1β, substance P, and VIP expression in the injured control DRG as compared to the contralateral uninjured DRG. In the ASC group, significant downregulation of inflammatory gene expression on the injured DRG as compared to the control group (data were presented with mean ± standard deviation, *indicated *p* < 0.05; **indicated *p* < 0.01, ***indicated *p* < 0.001, ****indicated *p* < 0.00001, ns indicated no significant difference; *n* = 3–5 for each group).

To explore the regulation of inflammatory signals in the early phase after ASC treatment, the regulatory genes involved in inflammatory signaling were analyzed in the injured nerve and the DRG using qPCR on day 3 after ASC injection. In the injured nerve, the expression levels of IL-1β, TNF-α, CD68, CD11b, CD80, and CD86 were all significantly upregulated in the control group, while they were downregulated in the ASC group (Figure 7A). Regarding the inflammatory signals of the ipsilateral DRG, upregulation of both TNF-α and IL-1β was demonstrated in the control group, but downregulation was demonstrated in the ASC group (Figure 7B). Moreover, prolonged upregulation of the pain-related genes, including vasoactive intestinal polypeptide (VIP) and substance P, were all significantly upregulated in the control group. The ASC group showed the downregulation of both VIP and substance P in the ipsilateral DRG on day 3 (Figure 7B).

## 4. Discussion

Stem cell therapy is a novel therapeutic approach for various neurological disorders. Clinical trials in the United States have investigated the functional benefits of stem cell therapy for multiple sclerosis, amyotrophic lateral sclerosis, Alzheimer's disease, Duchenne muscular dystrophy, Parkinson's disease, Huntington's disease, traumatic spinal cord injury, and other disorders of the central nervous system (Tabakow et al., 2013; Squillaro et al., 2016; Kubiak et al., 2020). However, there have been very few clinical trials examining the sensory and motor benefits of stem cells for traumatic peripheral nerve injury (De la Rosa et al., 2018; Kubiak et al., 2020). For compressive neuropathy, a recent clinical study demonstrated the safety and feasibility of transplantation of autologous fat derivates to nerves after their release from compression, with benefits for functional and sensory recovery (Krzesniak et al., 2021). However, the underlying mechanism and the local and remote modulation of neuroinflammation have not yet been investigated.

**Figure 8:**
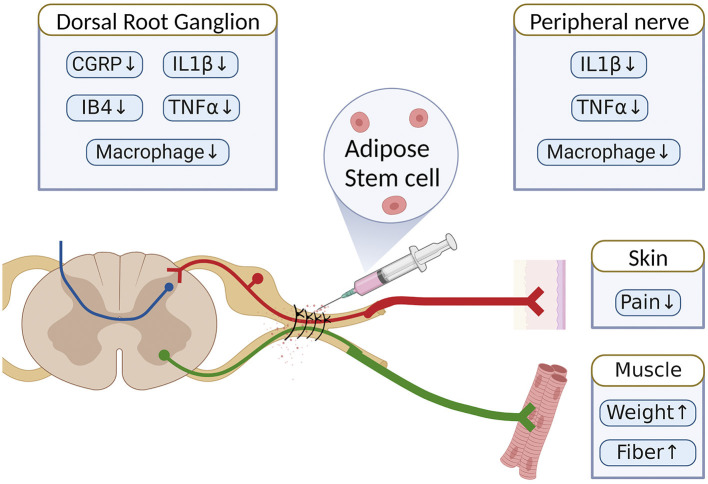
Schematic summary of the therapeutic benefit of ASCs for the alleviation of neuropathic pain in chronic constriction injury of rats.

In this study, the local treatment with ASCs significantly reduced neuropathic pain and muscle atrophy in irreversible peripheral compressive neuropathy. The beneficial sensory and motor outcomes were demonstrated by an increase in the innervated muscle's weight and muscle fiber size, a decrease in mechanical allodynia, and an improvement of electrophysiological results and gait function after ASC treatment (Figure 8). Venturi et al. (2015), reported that patients with pudendal neuralgia had a substantial decrease in pain scores after 1 year of treatment with transperineal injections of autologous ASC. Other studies have also demonstrated the neuroregenerative effects of ASC in the repair of peripheral nerves in both acute and chronic sciatic denervation injuries (Liu G. et al., 2011; Tomita et al., 2012). Local ASC therapy for rodent critical sciatic nerve gaps of more than 10 mm revealed beneficial effects on walking and gastrocnemius muscle regeneration (Liu G. B. et al., 2011; Hsueh et al., 2014). In addition, some investigators used electrophysiological assessments such as NCV and CMAP to investigate the functional recovery of the injured nerve after ASC therapy. They confirmed the benefits of local ASC treatment in terms of electrophysiological recovery (Vishnoi et al., 2019; Zhou et al., 2020). Our data provided identical results in the animal model of peripheral compressive neuropathy.

The success of stem cell therapy is significantly associated with the survival of ASCs in the injured nerve. However, stem cell therapy-induced tumorigenicity might be a potential risk, which positively correlates with the number of stem cells used. However, no studies have officially reported the optimal dosing of ASCs. Most of the studies have indicated that 1 × 10^6^ ASCs are needed to obtain a therapeutic effect (Wang et al., 2012; Rodriguez Sanchez et al., 2019; Jiang et al., 2022). To further investigate the regulatory effects of ASCs on the compressed nerve, the number of regenerated axons in the injured sciatic nerve was evaluated. Despite nerve release, immunofluorescent staining revealed significant axon demyelination by day 28. Local ASC treatment significantly enhanced the ratio of functional axon regeneration compared with the nerve release alone group, indicating the beneficial effect of local ASC therapy (Figures 2A, B). Similar findings were reported by Marconi et al. (2012) with functional axon regeneration after ASC therapy observed 6 weeks after sciatic nerve crush injury. Furthermore, prolonged neuroinflammation had been identified as significantly contributing to neuropathic pain in a CCI model from our previous reports (Chen et al., 2020, 2021). With local ASC treatment, the inflammatory cytokines (TNF-α, IL-1β) and infiltration of CD68+ inflammatory cells were significantly lower after ASC therapy than in the control group on day 28 (Figures 2C, D, 3). Excessive inflammation in the peripheral and central nervous systems may contribute to ectopic activity and central sensitization resulting from a robust immune response in developing refractory compressive neuropathy (Ellis and Bennett, 2013). Nerve damage leads to macrophage infiltration, T cell activation, and increased expression of proinflammatory cytokines, including the interleukins 1β, 6, 12, and 18, interferon-γ, tumor necrosis factor (TNF), and leukemia inhibitory factor (Tofaris et al., 2002). ASCs play an important role in regulating the proliferation and cytokine production of T cells in response to mitogens (Yañez et al., 2006; Gonzalez-Rey et al., 2010). They also decrease the production of inflammatory cytokines, such as TNF-α, IL-1β, and IL-6 (Premaratne et al., 2011; Franchi et al., 2014; Lee et al., 2015; Guo et al., 2016; Zhang et al., 2017). Improvements in neurological recovery in injured animals with ASC therapy might result from a local surge of growth factors that activate residential Schwann cells to secrete neurotrophic factors and facilitate axon myelination within the nerve (Ren et al., 2012; Dai et al., 2013). Moreover, several investigators have proposed an important influence of macrophage modulation on nerve regeneration after injury (Heo et al., 2019). Macrophages are abundant with multiple phenotypes that participate in nerve degeneration and regeneration in different ways (Gaudet et al., 2011). After nerve injury, circulating macrophages arrive at the site of the lesion within 1 day and peak within 2–3 weeks (Shen et al., 2000). The subsequent cellular events include phagocytosis, myelin debris removal, growth factor production, and remodeling of the extracellular matrix of the injured nerve (Griffin et al., 1993; Mueller et al., 2003). Pan-macrophage markers, such as CD68, indicate that macrophages infiltrate the injured nerve to induce phagocytosis, proinflammatory cytokines, and chemokine production (Stevens et al., 2019). Marconi et al., found that inflammatory cells including lymphocytes and macrophages are reduced after intravenous ASC treatment in an animal model of sciatic nerve crush injury (Marconi et al., 2012). These results were compatible with our study, based on the finding that local ASC treatment reduced macrophage infiltration relative to the control group. Our studies also demonstrated that ASCs modulate local neuroinflammation and promote functional recovery in rodent sciatic nerve compressive injury. In the injured nerve after ASC treatment on day 3, specific genes involving inflammatory macrophages (Kiguchi et al., 2018; Lim et al., 2021) such as CD68, CD80, and CD86 were all upregulated in the control group, while they were downregulated in the ASC group (Figure 7A). Overall, local ASC cell therapy promoted the improvement of neuropathology and the functional recovery of sensory and motor functions by the regulation of neuroinflammatory signals in the early phase after treatment.

Genetic expression changes associated with neuroinflammation, and neuropathic pain were observed within the DRG after peripheral nerve injury (Martin et al., 2019). Inflammatory cytokines, including TNF-α and IL-1β, recruited in the DRG after the nerve injury may eventually induce macrophage phagocytosis, Wallerian degeneration, and nerve regeneration (Fregnan et al., 2012). The neuropeptides substance P and CGRP have been widely shown to be involved in pain transmission after sciatic nerve injury and are dominantly expressed in the sensory neurons of the DRG (Fu et al., 2013). In addition, vasoactive intestinal peptide (VIP) in the DRG was upregulated, activating the mechanisms of neuropathic pain following peripheral nerve injury (Son et al., 2007; Woodley et al., 2019). Consequently, investigators aimed to apply stem cell therapy to reduce macrophage infiltration and inflammatory cytokines, thus relieving neuropathic pain (Siniscalco et al., 2011; Sacerdote et al., 2013; Vadivelu et al., 2013). In our study, persistent neuropathic pain was present in the control group but was relieved by ASC treatment (Figure 1B). Similarly, neuropathic pain markers, including CGRP and IB4, were induced in the DRG in the control group, while they were significantly lower in the ASC group (Figures 4A, B). The upregulation in substance P and VIP expression demonstrated a positive correlation with sustained induced mechanical allodynia (Figure 7B). Furthermore, CD68+ macrophages and neuroinflammatory cytokines TNF-α and IL-1β were extensively expressed in the DRG in the control group but showed significantly lower levels in the ASC group (Figures 4C, D, 5). In the ASC group, the local and remote modulation of neuroinflammation might indicate a promising treatment for extensive peripheral neuropathy.

A previous study demonstrated that the gene expression of both IL-1β and TNF-α was rapidly upregulated in the injured sciatic nerve and DRG, which was partly compatible with the results of this study (Figure 7) (Nadeau et al., 2011). However, the protein expression of TNF-α revealed no difference between groups in the DRG (Figures 5C, D). Several reports have indicated that the proinflammatory cytokines, TNF-α and IL-1β, induce the innate immune response. Both TNF-α and IL-1β signaling pathways lead to the activation of similar transcription factors (NF-κB, activator protein-1, AP-1, and CCAAT/enhancer-binding protein beta, C/EBPβ); however, knock-out of TNFα and IL-1β receptors produced different responses to known activators (lipopolysaccharide, LPS) of the innate immune response. The contradictory responses to LPS indicate that TNFα and IL-1β regulate different processes (Rothe et al., 1993; Glaccum et al., 1997; Ott et al., 2007). The specific postsynaptic adhesion molecules belonging to the IL-1 family were found to regulate synapse formation and stabilization. IL-1β has been shown to alter dendritic morphology by regulating the IL-1R1AcP(b)/IL-1R1APL1 complex through a c-Jun terminal kinase (JNK) pathway (Montani et al., 2017; Bodnar et al., 2018). Further investigation is needed to differentiate the influences of TNFα and IL-1β signaling in the DRG and the remote immunoregulatory effect of ASC therapy.

The therapeutic mechanisms of transplanted ASCs remain unclear and complicated. Two theoretical mechanisms were proposed from previous studies (Jiang et al., 2022). One potential benefit is that ASCs could facilitate peripheral nerve repair and regeneration through structural support and *in vivo* differentiation in injured neural tissue (Tomita et al., 2013). However, other investigators found no evidence of *in vivo* transdifferentiation of ASCs into Schwann cells after transplantation (Santiago et al., 2009; Marconi et al., 2012). Another proposed mechanism is that ASCs may promote nerve regeneration by secreting nerve growth factors and releasing paracrine signals to enhance vascularization, preventing tissue necrosis, or suppressing immune responses (Marconi et al., 2012; Bucan et al., 2019). To investigate the terminal fate and beneficial mechanisms of the transplanted ASC in the injured nerve, we tracked the ASCs during the first week after injection. Interestingly, the bioluminescent signals remained active on days 1 and 3 but declined to background levels on day 7 (Figures 6A, B). The functional phenotype of transplanted ASCs was maintained within the local injured nerve on day 3, as demonstrated by the coexpression of CD90 and CD105 in the injured nerve (Figure 6C). The brief period of cell survival revealed that most of the transplanted cells were lost before full functional recovery took place, which correlated with previous studies (Walsh and Midha, 2009; Erba et al., 2010; Walsh et al., 2012). Accordingly, the beneficial effect of locally transplanted ASCs on nerve regeneration and reduction of neuroinflammation might result from local and remote paracrine modulation in the compressed nerve. In addition to surgical decompression, ASC therapy has the potential clinical benefits of alleviating neuropathic pain by promoting nerve regeneration, neurophysiologic conduction, and immunomodulation (Joshi et al., 2021; Bryk et al., 2022; Lee et al., 2022).

This study validates that local ASC therapy promotes functional recovery in the irreversible compressive injury of peripheral nerves. The major limitations of this study were as follows: First, the induced mechanical allodynia from the CCI model was not identical to neuropathic pain in clinical patients with compressive neuropathy. Therefore, the clinical application of local stem cell therapy should be considered with caution. Second, this research only demonstrated a short-term beneficial mechanism of stem cell therapy from allogenic sources. The long-term cell fate of autologous stem cell transplantation should be further investigated for clinical translation.

## 5. Conclusion

Compressive neuropathy remains a clinical challenge in terms of residual neuropathic pain and irreversible dysfunction. Repeated decompression, variable medication, and physical therapy provide unsatisfactory benefits and may cause unnecessary injury or complications. ASCs, with their neuroregenerative and immunomodulatory properties, may provide promising local and remote beneficial influences that alleviate neuropathic pain and promote functional recovery of compressive neuropathy. In this study, the locally delivered ASC therapy after a nerve decompression procedure provided additional therapeutic benefits for sensory and motor recovery in the rodent sciatic nerve after CCI. Stem cell therapy contributes to alleviating neuropathic pain, reducing neuroinflammation, and inhibiting inflammatory macrophage recruitment at the site of peripheral nerve injury and in the involved DRG. With these advances, local ASC therapy may offer a novel and promising treatment for clinically recurrent or intractable compressive neuropathy.

## Data availability statement

The datasets presented in this study can be found in online repositories. The names of the repository/repositories and accession number(s) can be found in the article/Supplementary material.

## Ethics statement

The studies involving human participants were reviewed and approved by Institutional Review Board (IRB, approval number A-ER-109-127) of the National Cheng Kung University Hospital (NCKUH). The patients/participants provided their written informed consent to participate in this study. The animal study was reviewed and approved by Laboratory Animal Center and Institutional Animal Care and Use Committee (IACUC, Approval number 109250) at National Cheng Kung University (Tainan, Taiwan).

## Author contributions

S-HC and W-LT performed experiments and collected the data. C-CW, S-CL, and Y-YH developed the concepts and designed the experiments. F-IL, Y-HL, and S-PL provided system and technical support, discussion, and problem-solving in research. S-HC and Y-YH wrote the manuscript. S-CL and Y-YH revised the manuscript and organized the current study. All authors have read and agreed to the published version of the manuscript.

## Supplementary material

Supplementary Figure 1 Characteristics of adipose-derived stem cells. **(A)** Cell markers of ASCs analyzed by flow cytometry revealed that the majority of the injected cells were CD34^−^, CD90^+^, and CD105+ cells. **(B)** The multipotent potential of ASC demonstrated the trilineage differentiation ability into osteogenic, adipogenic, and chondrogenic lineages (Scale bar: 100 μm).

Supplementary Figure 2 *In vitro* validation of ICG ASC tracing. ICG-labeled ASCs were able to track bioluminescent signals *in vitro* for more than 7 days using the non-invasive IVIS Spectrum System.
