# Supplementary figures and images for: Adipose-derived stem cells modulate neuroinflammation and improve functional recovery in chronic constriction injury of the rat sciatic nerve
Source: Front Neurosci. 2023 Jun 29;17:1172740. doi: 10.3389/fnins.2023.1172740 (PMC10339833; doi:10.3389/fnins.2023.1172740)

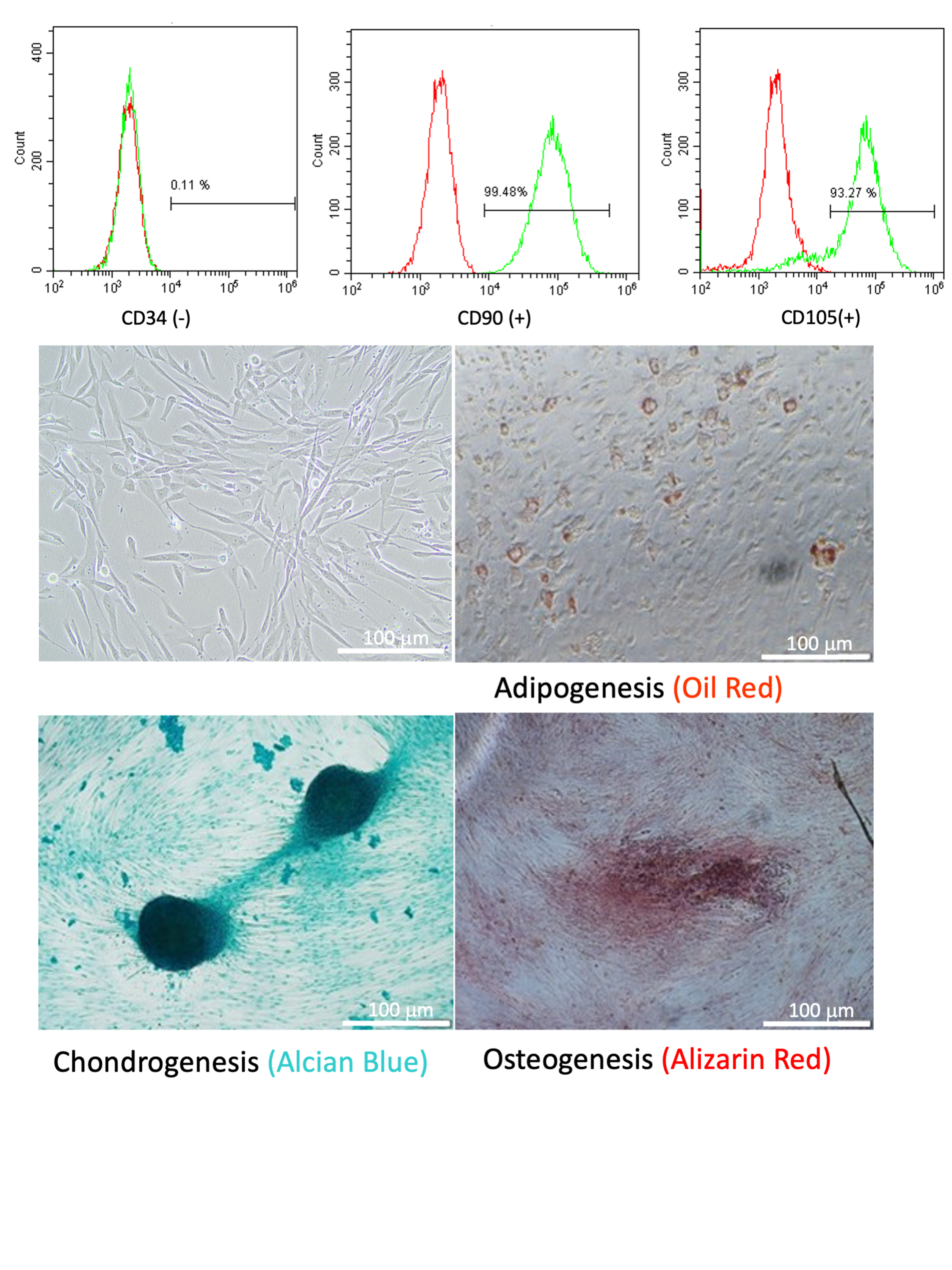

Supplement: Supplementary Figure 1 — Characteristics of adipose-derived stem cells. (A) Cell markers of ASCs analyzed by flow cytometry revealed that the majority of the injected cells were CD34−, CD90+, and CD105+ cells. (B) The multipotent potential of ASC demonstrated the trilineage differentiation ability into osteogenic, adipogenic, and chondrogenic lineages (Scale bar: 100 μm). [file Image_1.TIFF]

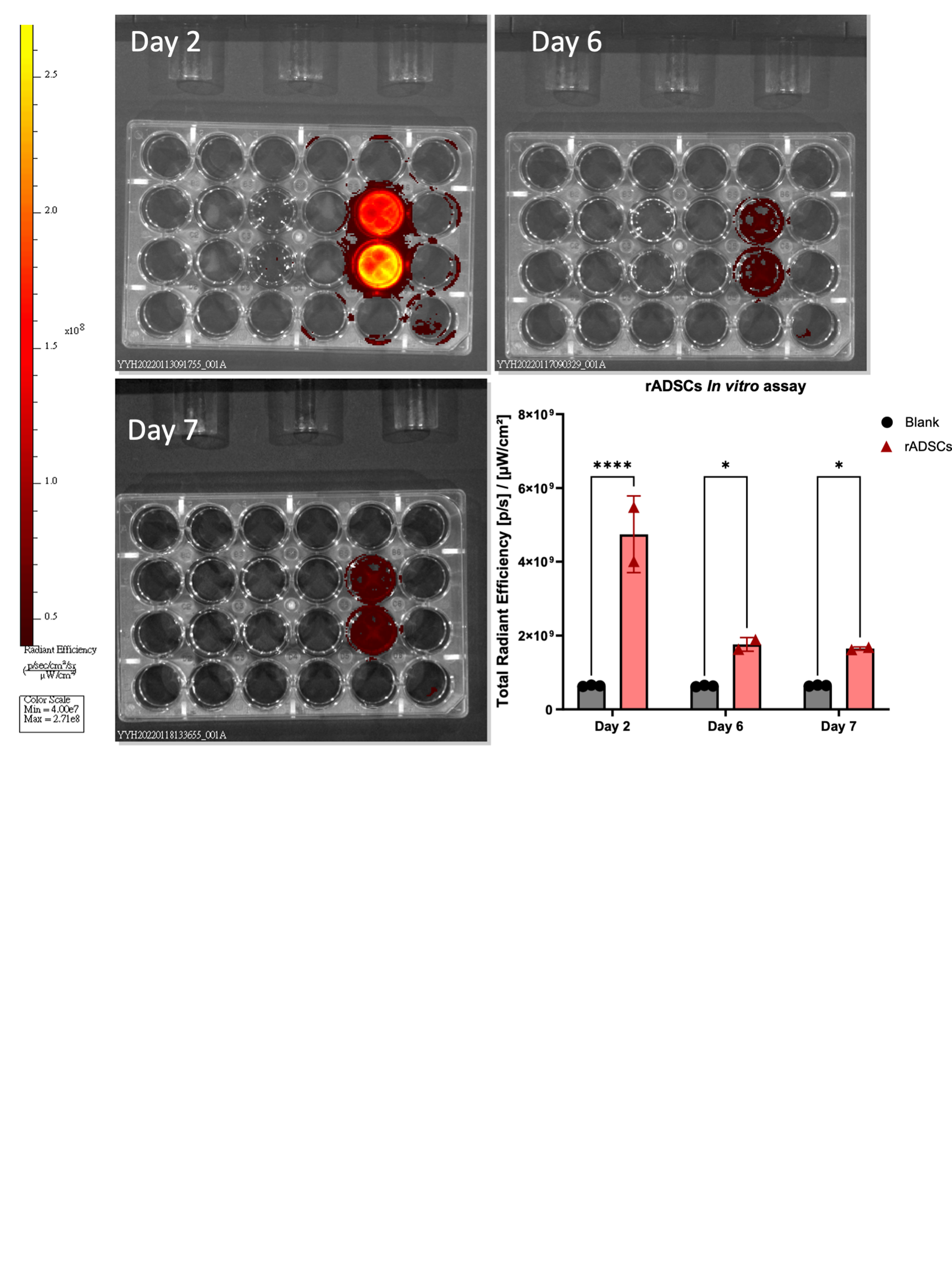

Supplement: Supplementary Figure 2 — In vitro validation of ICG ASC tracing. ICG-labeled ASCs were able to track bioluminescent signals in vitro for more than 7 days using the non-invasive IVIS Spectrum System. [file Image_2.TIFF]
