# Supplementary material for: Adipose-derived stem cells modulate neuroinflammation and improve functional recovery in chronic constriction injury of the rat sciatic nerve
Source: Front Neurosci. 2023 Jun 29;17:1172740. doi: 10.3389/fnins.2023.1172740 (PMC10339833; doi:10.3389/fnins.2023.1172740)
Supplement: Supplementary file 3 [file Table_1.DOCX]

Supplementary Table 1

|  | Adipose stem cell |
| --- | --- |
| Passage Number | P4-P5 |
| Transplanted number per nerve | 1 × 10^6^ |
| Cell survival | >90% |
| Surface antigen (flow cytometry) |  |
| CD90 | 99.48% |
| CD105 | 93.27% |
| CD73 | 98.2% |
| CD34 | 0.11% |
| CD45 | 1.1% |
| HLA-DR | 0.4% |
| CD11b | 0.2% |
